# Introduction of a cascaded segmentation pipeline for parametric T1 mapping in cardiovascular magnetic resonance to improve segmentation performance

**DOI:** 10.1038/s41598-023-28975-5

**Published:** 2023-02-06

**Authors:** Darian Viezzer, Thomas Hadler, Clemens Ammann, Edyta Blaszczyk, Maximilian Fenski, Thomas Hiroshi Grandy, Jens Wetzl, Steffen Lange, Jeanette Schulz-Menger

**Affiliations:** 1 ECRC Experimental and Clinical Research Center, Charité – Universitätsmedizin Berlin, corporate member of Freie Universität Berlin and Humboldt-Universität zu Berlin, Lindenberger Weg 80, 13125 Berlin, Germany; 2 Working Group on Cardiovascular Magnetic Resonance, Experimental and Clinical Research Center, a joint cooperation between the Charité – Universitätsmedizin Berlin and the Max-Delbrück-Center for Molecular Medicine, Berlin, Germany; 3 DZHK (German Centre for Cardiovascular Research), Partner Site Berlin, Berlin, Germany; 4 Department of Cardiology and Nephrology, Helios Hospital Berlin-Buch, Berlin, Germany; 5 Siemens Healthcare GmbH, Erlangen, Germany; 6 Faculty for Computer Sciences, Hochschule Darmstadt (University of Applied Sciences), Darmstadt, Germany

**Keywords:** Cardiology, Mathematics and computing, Software

## Abstract

The manual and often time-consuming segmentation of the myocardium in cardiovascular magnetic resonance is increasingly automated using convolutional neural networks (CNNs). This study proposes a cascaded segmentation (CASEG) approach to improve automatic image segmentation quality. First, an object detection algorithm predicts a bounding box (BB) for the left ventricular myocardium whose 1.5 times enlargement defines the region of interest (ROI). Then, the ROI image section is fed into a U-Net based segmentation. Two CASEG variants were evaluated: one using the ROI cropped image solely (cropU) and the other using a 2-channel-image additionally containing the original BB image section (crinU). Both were compared to a classical U-Net segmentation (refU). All networks share the same hyperparameters and were tested on basal and midventricular slices of native and contrast enhanced (CE) MOLLI T1 maps. Dice Similarity Coefficient improved significantly (p < 0.05) in cropU and crinU compared to refU (81.06%, 81.22%, 72.79% for native and 80.70%, 79.18%, 71.41% for CE data), while no significant improvement (p < 0.05) was achieved in the mean absolute error of the T1 time (11.94 ms, 12.45 ms, 14.22 ms for native and 5.32 ms, 6.07 ms, 5.89 ms for CE data). In conclusion, CASEG provides an improved geometric concordance but needs further improvement in the quantitative outcome.

## Introduction

Cardiovascular magnetic resonance (CMR) is one of the most important non-invasive imaging modalities for risk stratification in cardiovascular diseases^1,2^. It enables the characterization of focal and diffuse changes in the myocardial tissue by quantitative techniques such as parametric mapping^3^, which is considered as one of the most meaningful innovations in recent CMR developments^4,5^. While T2 mapping is used for the detection of myocardial edemas, T1 mapping is applied across multiple pathophysiological mechanisms and tissue characteristics^4^. A T1 map can be acquired before, referred as native, or after application of a contrast agent, referred to as contrast enhanced (CE)^6^. The latter is usually integrated in the calculation of an extracellular volume (ECV) map.

For diagnostics, the post-processing involves the segmentation of the myocardium as tissue of interest in order to obtain quantitative values^7^. This is usually performed in a manual and often time consuming manner^1,7–10^. Furthermore, even experienced readers show intra-observer variability that results in considerable quantification uncertainty^11^. Current developments attempt to overcome both, the time consumption and the reproducibility uncertainty by using convolutional neural networks (CNNs) to automatically segment the tissue of interest. One of the most prominent CNN models in medical segmentation tasks is the U-Net^1^ introduced by Ronneberger et al.^12^. While a standard U-Net already shows an average geometrical overlap above 70% with an expert segmentation^8–10,13^, which is conventionally assumed as a good result^8^, current development focuses on technical improvements for even better segmentation quality. At present, many strategies intend to advance the CNN models towards more complex framework structures^8,9^ or integrating alternative architecture structures^14^.

Parametric maps are images with pixel values representing the amplitude of a physical quantity. However, the vast majority of pixels contain superfluous background information. Object detection algorithms (ODAs) are used to find object regions of interest (ROI) in images by localizing a bounding box (BB) around those objects with the help of CNNs^15,16^. Consequently, ODAs can help to focus on a ROI in parametric mapping and thus, to reduce the amount of background information that is fed into an automatic segmentation network.

Although the detection of left ventricular myocardium by ODAs already exist for CINE images in CMR^16^, its application on parametric T1 mapping and combination with automatically segmenting CNN procedures remains to the best of our knowledge unaddressed. Consequently, the aim of this study is to analyze the impact of input data enhancement on the segmentation quality in parametric T1 mapping by introducing an ODA as a preliminary processing step before the actual segmentation task. This coarse to fine segmentation procedure is named in the following as cascaded segmentation (CASEG).

## Materials and methods

### Dataset

A heterogeneous dataset of parametric T1 maps with corresponding manual reference segmentation from published^17–19^ and on-going studies^20^ was used. The inline T1 maps were either generated on a 1.5 T AvantoFit, a 3 T SkyraFit or a 3 T PrismaFit clinical magnetic resonance imaging scanner (all Siemens Healthcare, Erlangen, Germany) and were based on the MOLLI sequence using a 5(3)3 scheme for native and a 4(1)3(1)2 scheme for CE acquisitions.

Data from N = 403 participants (97 healthy volunteers and 306 patients) were used resulting in a total of M = 1438 parametric T1 maps, of which 1080 were native and 358 CE T1 maps. The difference in the quantity of native compared to CE T1 maps is due to the absence of CE measurements in some of the original studies. The dataset was randomly split per study set into 75% training, 10% validation and 15% test data. Table 1 shows an overview of the dataset and the amount for training, validation and testing. While some source studies in the dataset contained a full short axis T1 map stack, others only had three (basal, midventricular and apical), two (mostly basal and midventricular) or solely one (mostly midventricular) slice. The training and validation were done on all assigned T1 maps to assure for an advanced generalization of the segmentation network, whereas, the test dataset was restricted to midventricular and basal slices only as recommended by the society for cardiovascular magnetic resonance^4^. The reference segmentation was performed manually by experienced readers using the software cvi42 (Circle Cardiovascular Imaging, Calgary, Canada). The data acquisition and manual segmentation processing were performed in accordance with relevant guidelines and regulations. This study was approved by the local ethics committee of the Charité Universitätsmedizin Berlin (study ID: EA 1 253 21).

**Table 1:** Overview of the complete dataset, the (numbers) in brackets denotes the number for midventricular and basal slices only that are used as test dataset in this study.

	Training	Validation	Testing	Total
Subjects (N)	313	35	55 (55)	403
Native T1 maps	849	91	140 (106)	1080
Contrast enhanced T1 maps	286	27	45 (33)	358
Total T1 maps (M)	1135	118	185 (139)	1438

### ODA

A CNN based ODA was used to detect a BB^15^ that tightly fits the left ventricular myocardium in the parametric T1 map. During training, the ODA CNN behaved like a conventional segmentation CNN by providing the target BB data as binary mask. The predicted raw output of the ODA CNN model, in turn, needed a postprocessing in order to represent a binary mask of a BB. First, the output was thresholded at a value of 0.5, then the largest connected component (LCC) was identified as the BB ROI. The minimum and maximum indeces of the LCC along both image axis defined the BB edges. These edges were finally converted into a BB binary mask.

A magnification factor to enlarge the BB was evaluated in order to securely cover the whole left ventricle within the ROI. The maximum occurring factor across the test data is assumed as suitable to guarantee for this. This factor was used to compensate uncertainties from the CNN based prediction while keeping the ROI small compared to the original image size.

### CASEG

The basic idea of CASEG was the subsequent arrangement of two independent CNN models. The ODA served as a first coarse prediction of a potential ROI while the second CNN was applied on the focused image section in the ROI for the actual segmentation task and returned the final segmentation mask.

**Figure 1:**
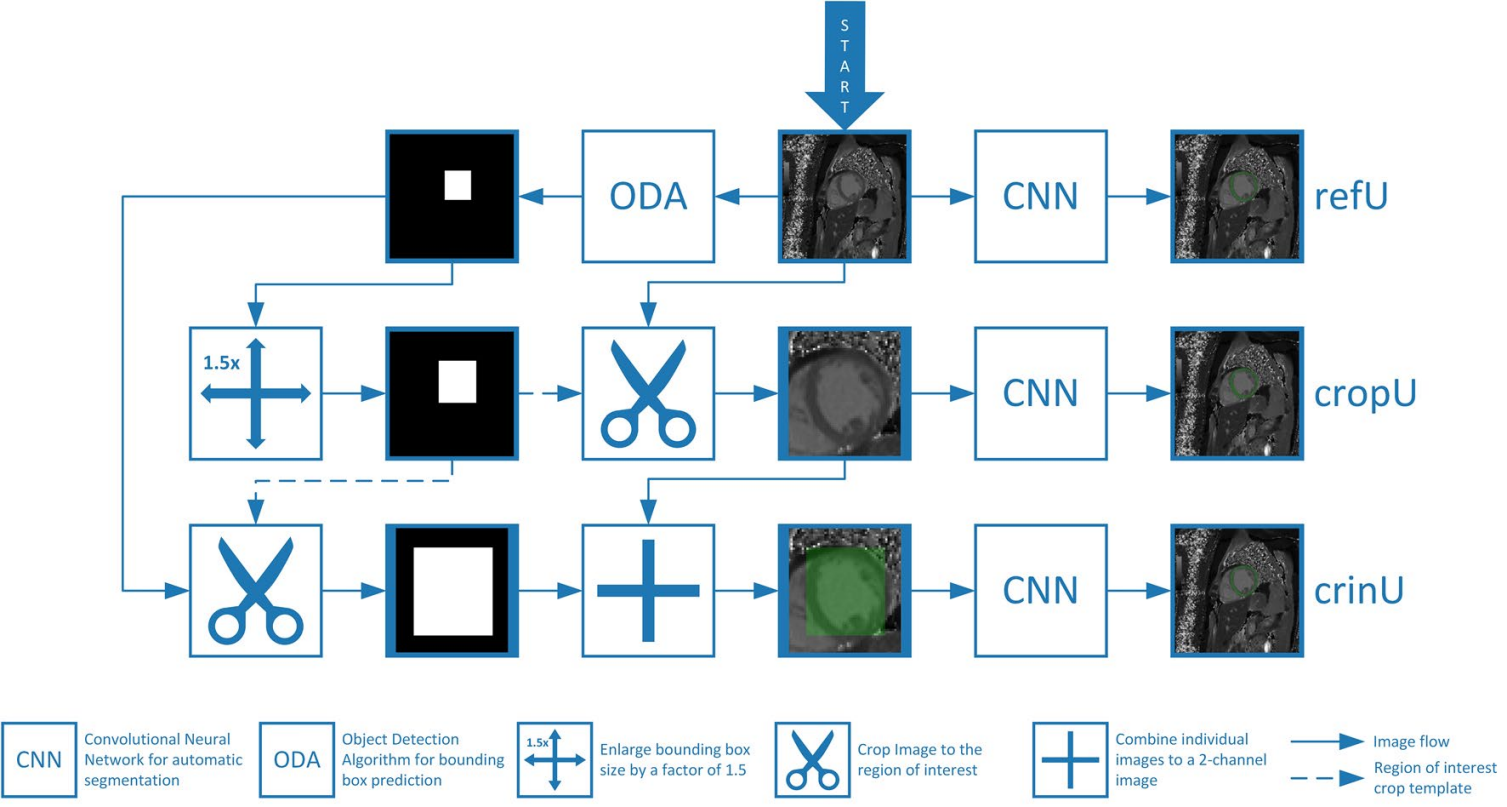
Processing pipelines for refU, cropU and crinU. Convolutional neural networks (CNNs) are used for the segmentation of the myocardium as tissue of interest. While refU directly uses the input image, cropU and crinU use the region of interest image section that belongs to the 1.5 times enlarged bounding box from an object detection algorithm (ODA). In contrast to cropU, crinU uses a two channel image with the second channel having the original predicted bounding box mask.

Due to the BB enlargement for the ROI definition, two potential CASEG pipelines (cropU and crinU) were evaluated as visualized in Fig. 1. In cropU, the input image for the secondary segmentation CNN was the original image cropped to the ROI image section. In contrast to that, the crinU worked in a similar manner and also considered the cropped image section. However, in crinU, the input image for the secondary segmentation CNN was extended by a second channel that comprises the unenlarged BB (shown as green overlay in Fig. 1). Considering cropU, an alternative implementation exists as visualized in Supplemental Material S1 by having an ODA that directly predicts an enlarged BB without the magnification factor processing step between ODA and segmentation network (cropU_A).

All three CASEG pipelines were compared to a reference pipeline (refU) that was equivalent to the secondary segmentation model without any preliminary ODA such that the original image was directly fed into the CNN.

### Quality assurance

All CNN models including the ODA were implemented as U-Nets. As this study aimed to analyze the impact of input data enhancement by using a preliminary ODA, the same hyperparameters were used for all CNNs except for the number of input channels. The selected hyperparameters showed the best results in the reference refU. The models had 27 layers with 6 skip connections and the input size was 256 × 256 × 2 for the secondary segmentation model in crinU and 256 × 256 × 1 for all other U-Net models. A detailed U-Net structure overview is shown in the Supplemental Material S2. Consequently, the input images were resized and interpolated to the model specific size. The log-cosh-dice loss function^21^ with an Adam optimizer^22^ having a clipnorm of 0.001 was used. The batch size was set to 10 and the number of epochs was 1000 but an early stopping scheme^23^ that ended the training after 50 epochs of no improvement with respect to the dice similarity coefficient metric in the validation data was used. The learning rate, which reflected the maximum learning rate value the Adam optimizer could capture, of initially 0.001 was halved every 25 epochs of no improvement.

During the training of the individual models, the training dataset was randomly augmented^24^ with brightness adjustments, contrast adjustments, blurring, Gaussian random noise, salt and pepper noise, rotation, mirroring, axis downsampling and, for the refU and ODA only, a cropping of the image. Additionally, during training of the secondary segmentation CNN models in cropU and crinU, the detected BB was randomly shifted and resized by up to 5 pixels, whereas in 5% the optimal BB was used instead of the predicted one and in another 5% a failed BB detection was assumed in order to reflect potential detection uncertainties.

In case of a BB prediction failure, the ODA returned a binary image with only zero values reflecting no found BB. In such a case, cropU behaved similar to refU and used the original image as input. Further, crinU used the original image as first channel as well and kept the second channel zero valued. If the BB enlargement exceeded the image boundaries, the ROI was cropped at that boundary such that it never exceeded the original image.

For normalization of the input images, each input image channel was scaled to floating point values between zero and one. Further, while the input images were internally resized to the model specific size, the output was back transformed to the original input image size. To losslessly apply this and other geometric transformations, the segmentation masks were converted from binary pixel masks to vectorized contour objects allowing for geometrically precise transformation. The transformed structures were then rasterized back into pixel masks.

The primary domain of the output quality measure is of geometric nature. The geometric domain reflects the spatial similarity of two individual segmentations. For that reason, the Dice Similarity Coefficient (DSC) and the Hausdorff Distance (HD) were used as geometrical quality metrics. Both were supported visually with boxplots. As the DSC and HD were not normally distributed, non-parametric Friedman (across all models) and Wilcoxon (across refU and either CASEG pipeline) tests were used with a significance level of p < 0.05. Significance was assumed if both statistical tests were significant. Additionally, the enlarged BB detection was tested for the increment in the ratio of relevant pixels reflecting the foreground information to the total number of pixels in the image section compared to the original image. The ratio increment was tested with a Wilcoxon test for significance with a significance level of p < 0.05.

As parametric T1 maps provide clinically interpretable quantitative measurements, the quantitative domain was tested for the effect of the four segmentation approaches on the estimated average T1 time. The mean error (ME), mean absolute error (MAE) and root mean squared error (RMSE) were used in combination with the confidence interval (CI) to evaluate the quality in the quantitative domain. The CI were tested for remaining within the published equivalence margin derived from an intra-observer variability of native T1 maps, which is defined as the clinically acceptable deviation^11^. As the four models were evaluated on the same test dataset, the CIs were Bonferoni corrected^11^. The coefficient of variation (CV) with respect to the quantitative T1 error were additionally provided in order to analyze if the CE segmentations would remain in an adequate equivalence margin for CE data.

As the ME and MAE are not normally distributed, non-parametric Friedman and Wilcoxon tests were used for the comparison of these metrics with a significance level of p < 0.05. A lower RMSE was assumed to indicate an improvement.

Additionally, a correlation plot including linear regression, the Pearson Correlation (testing for linearity) and Kendall’s Tau (testing for rank-order stability) were provided. While conventionally correlation coefficient values are assumed as weak if smaller than 0.35, moderate if up to 0.67, strong if up to 0.90 and very strong if above, the coefficient of determination (CoD, squared Pearson correlation coefficient), represents the amount of shared variance between two measures and thus may support the interpretation of the findings^25^. The correlation plot was complemented with a Bland–Altman plot in order to visualize the limits of agreement^26^.

As T1 times of native and CE maps are on different scales, all analysis were separately performed on native and CE data.

### Source code

All programming tasks were implemented in Python (version 3.8, Python Software Foundation, Beaverton, USA). The necessary Python libraries with their specific used version are listed in the Supplemental Material S3. The U-Nets were implemented using the Tensorflow^27^ library (version 2.7.0) while statistics were calculated with the scipy^28^ package (version 1.4.1) and plots were created with the matplotlib^29^ library (version 3.5.2). The software includes a README file with a description for using the software. The user does not need to take care about processing between the ODA and the segmentation network as this is done automatically in the provided scripts.

### Ethical approval

This study was approved by the local ethics committee of the Charité Universitätsmedizin Berlin as retrospective study (study ID: EA 1 253 21). The requirement for written informed consent was acquired during the original clinical studies and was therefore waived in this study due to its retrospective design as approved by the local ethics committee of the Charité Universitätsmedizin Berlin (study ID: EA 1 253 21).

## Results

Numbers in the results are shown as mean ± standard deviation with their specific unit of measure.

### ODA

**Figure 2:**
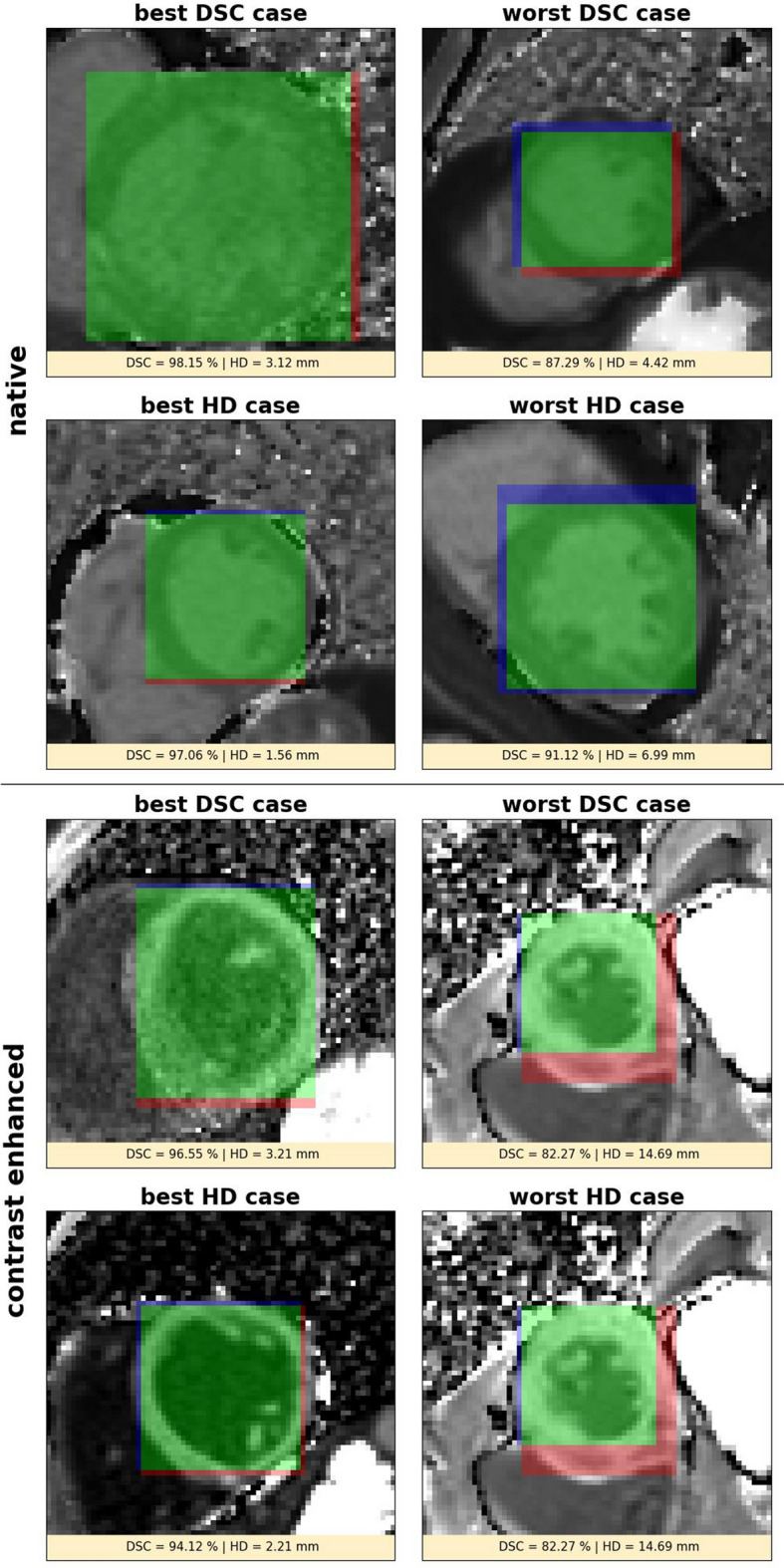
Example results of the object detection algorithm showing bounding boxes for the left ventricular myocardium. The upper block corresponds to native and the lower block to contrast enhanced data; respectively in each block the first row corresponds with respect to the Dice Similarity Coefficient (DSC) and the second row corresponds with respect to the Hausdorff Distance (HD) while the first column shows the best and the second column the worst case. Green denotes true positive, blue false negative and red false positive segmented bounding box pixels.

The U-Net based ODA identified a BB of the left ventricle in all cases of the test dataset. Figure 2 shows respectively the best and worst cases for the BB prediction in regard of DSC and HD across native and CE test data. Throughout the whole test dataset, the predicted BBs resulted in a DSC of 93.09 ± 2.13% and 91.99 ± 2.80% and a HD of 3.95 ± 1.07 mm and 4.42 ± 2.40 mm for native and CE T1 maps respectively.

In order to securely cover the whole left ventricle with the BB across the test dataset, a magnification factor of at least 1.44 was necessary. Hence, cropU and crinU were set up with a rounded-up magnification factor of 1.50. In the Supplemental Material S4 the impact of the magnification factor on the average DSC results in the test dataset for cropU and crinU is shown. For magnification factors between 1.3 and 2.5, the results reached a performance plateau with minor fluctuations due to model training uncertainties.

Comparing the ratio of relevant foreground pixels to the total number of pixels, the ratio increased significantly (p < 0.05) to10.38 ± 3.27% and 10.76 ± 4.22% in the test dataset for native and CE maps as compared to 0.71 ± 0.35% and 0.62 ± 0.21% when using the ROI image section instead of the original image. Assuming an unenlarged perfectly fitting BB, the maximum reachable ratio would be 20.12 ± 6.67% and 19.78 ± 6.00% for native and CE test data respectively. In the training and validation dataset, the increment was similar. Detailed boxplots of the ratio of relevant pixels are provided in the Supplemental Material S5 separately for native and CE data in the training, validation and test datasets.

### CASEG

**Figure 3:**
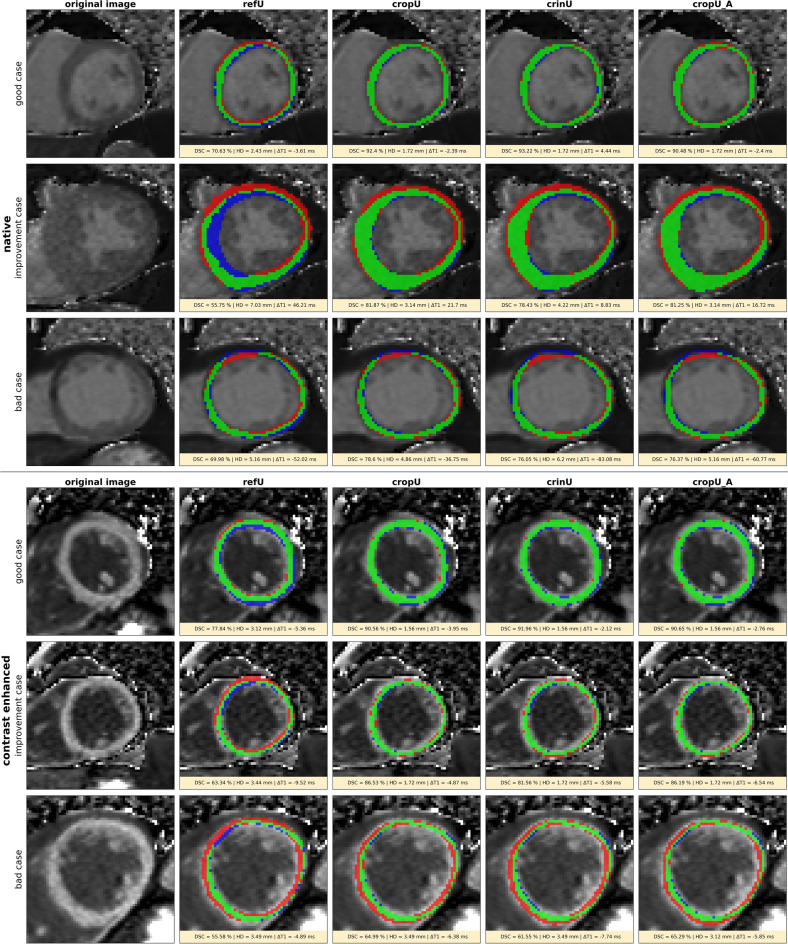
Example results of the automated segmentation in refU, cropU, crinU and cropU_A. The first column shows the original image, the second column the refU segmentation, the third column the cropU segmentation, the fourth column the crinU and the fifth column the cropU_A segmentation. The upper block corresponds to native and the lower block to contrast enhanced data; respectively in each block the first row shows a fairly good case across all four pipelines, the second row shows a case that is improved in cropU and crinU compared to refU and the third row shows a poor case across all four pipelines. Green denotes true positive, blue false negative and red false positive segmented pixels.

Exemplary segmentation results for refU, cropU, crinU and cropU_A are shown in Fig. 3 with a good case across all four pipelines, a case improving in CASEG compared to refU and a rather poor case across all four pipelines respectively for native and CE T1 maps. In Table 2 detailed results of geometric and quantitative metrics are provided.

**Table 2:** Overview of the geometric and quantitative results for refU, cropU, crinU and cropU_A in the T1 map test dataset separated for native and contrast enhanced T1 maps.

Metric	refU	cropU	crinU	cropU_A
**Native**				
*Geometric*				
DSC (%)	72.79 ± 8.08	81.06 ± 5.57*	81.22 ± 5.52*	81.13 ± 5.83*
HD (mm)	3.74 ± 1.37	2.95 ± 1.06*	3.01 ± 1.20*	2.98 ± 1.16*
*Quantitative*				
ME (ms)	− 7.22 ± 17.19	− 6.00 ± 14.67	− 5.24 ± 16.40*	− 3.88 ± 16.10*
MAE (ms)	14.22 ± 12.06	11.94 ± 10.43	12.45 ± 11.89	12.45 ± 11.89
RMSE (ms)	18.64	15.85	17.22	16.56
CI (ms)	− 11.38 / − 3.05	− 9.56 / − 2.44	− 9.22 / − 1.26	− 7.79 / 0.02
CV (%)	2.38	2.45	3.13	4.15
r (Pearson)	0.97*	0.97*	0.97*	0.97*
CoD (%)	94.09	94.09	94.09	94.09
τ (Kendall)	0.80*	0.83*	0.82*	0.83*
**Contrast enhanced**				
*Geometric*				
DSC (%)	71.41 ± 8.54	80.70 ± 10.31*	79.18 ± 10.20*	80.15 ± 10.21*
HD (mm)	3.83 ± 1.44	3.08 ± 1.72*	3.35 ± 1.90*	3.27 ± 2.05*
*Quantitative*				
ME (ms)	5.23 ± 8.14	4.45 ± 8.39	5.17 ± 7.27	4.57 ± 7.85
MAE (ms)	5.89 ± 7.67	5.32 ± 7.87	6.07 ± 6.54	5.07 ± 7.53
RMSE (ms)	9.67	9.50	8.92	9.08
CI (ms)	1.70 / 8.77	0.81 / 8.10	2.01 / 8.34	1.15 / 7.98
CV (%)	1.56	1.89	1.41	1.72
r (Pearson)	0.98*	0.98*	0.98*	0.98*
CoD (%)	96.04	96.04	96.04	96.04
τ (Kendall)	0.91*	0.91*	0.92*	0.94*

*DSC* dice similarity coefficient, *HD* Hausdorff distance, *ME* mean error, *MAE* mean absolute error, *RMSE* root-mean-squared error, *CI* confidence interval, *CV* coefficient of variation, *r* Pearson’s correlation coefficient, *CoD* coefficient of determination, *τ* Kendall’s Tau coefficient; values are given as mean ± standard deviation.

*Denotes statistical significance with a significance level of p < 0.05.

**Figure 4:**
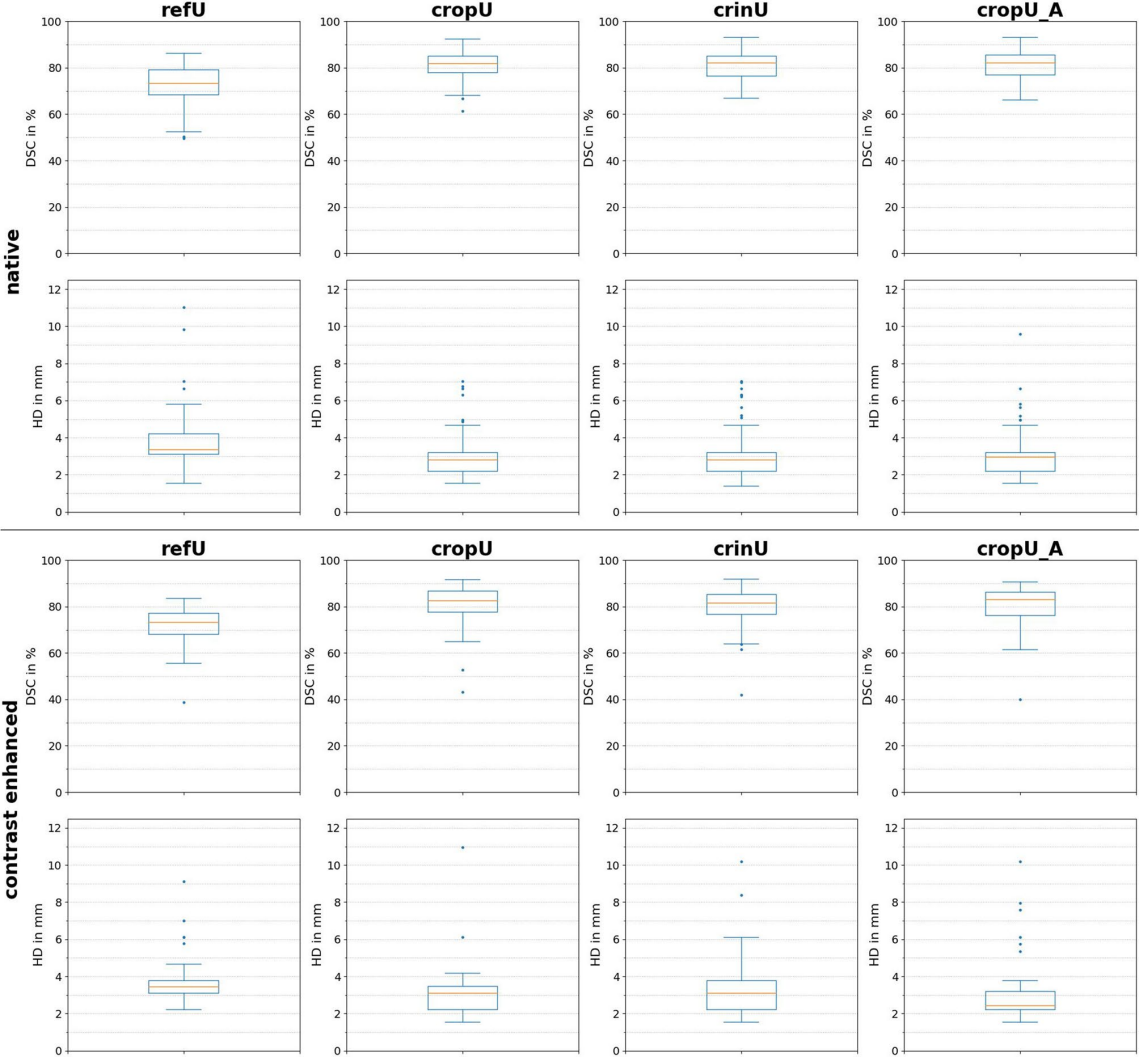
Geometric results of the automated segmentation. The first column shows the geometric results for refU, the second column for cropU, the third column for crinU and the fourth column for cropU_A. The upper block corresponds to native and the lower block to contrast enhanced data; respectively in each block the first row shows the boxplots of the Dice Similarity Coefficient (DSC) and the second row shows the boxplots of the Hausdorff Distance (HD).

The geometric quality improved significantly for all CASEG pipelines in comparison to the refU across the native and CE test datasets. The DSC improved significantly (p < 0.05) from around 72% towards 80% while the HDs were minimized significantly (p < 0.05) from above 3.70 mm to around 3.00 mm in all CASEG pipelines with the exception of a significant (p < 0.05) reduction to only 3.35 mm and 3.27 mm for CE data in crinU and cropU_A respectively. The corresponding boxplots in Fig. 4 illustrate these results and show that in some cases even a DSC of more than 90% was achieved in all three CASEG and across native and CE data, while refU reached a maximum DSC of 86.38% in native and 83.56% in CE data only. On the other hand, given 70% as threshold conventionally assumed as a good DSC^8^, there were still cases left in cropU, crinU and cropU_A below that margin. The HD showed a minimization in the CASEG but remained with an average distance of 2.95 mm to 3.35 mm within the range of two to three pixels deviation.

Numerically ME, MAE and RMSE were consistently reduced in cropU, crinU and cropU_A compared to refU in the native as well as CE test data except for MAE in crinU for CE test data as shown in Table 2. Neither ME nor MAE showed a significant (p < 0.05) improvement compared to refU except for ME in crinU and cropU_A for native data. The CI of all pipelines stayed within the equivalence margin of 24.5 ms^11^. A visual support of this result is shown in the Supplemental Material S6. The CV of the CE test data stayed below the CV of the corresponding native test data in all pipelines. As a consequence, the CE results were assumed to remain in an adequate equivalence range based on CE data only.

**Figure 5:**
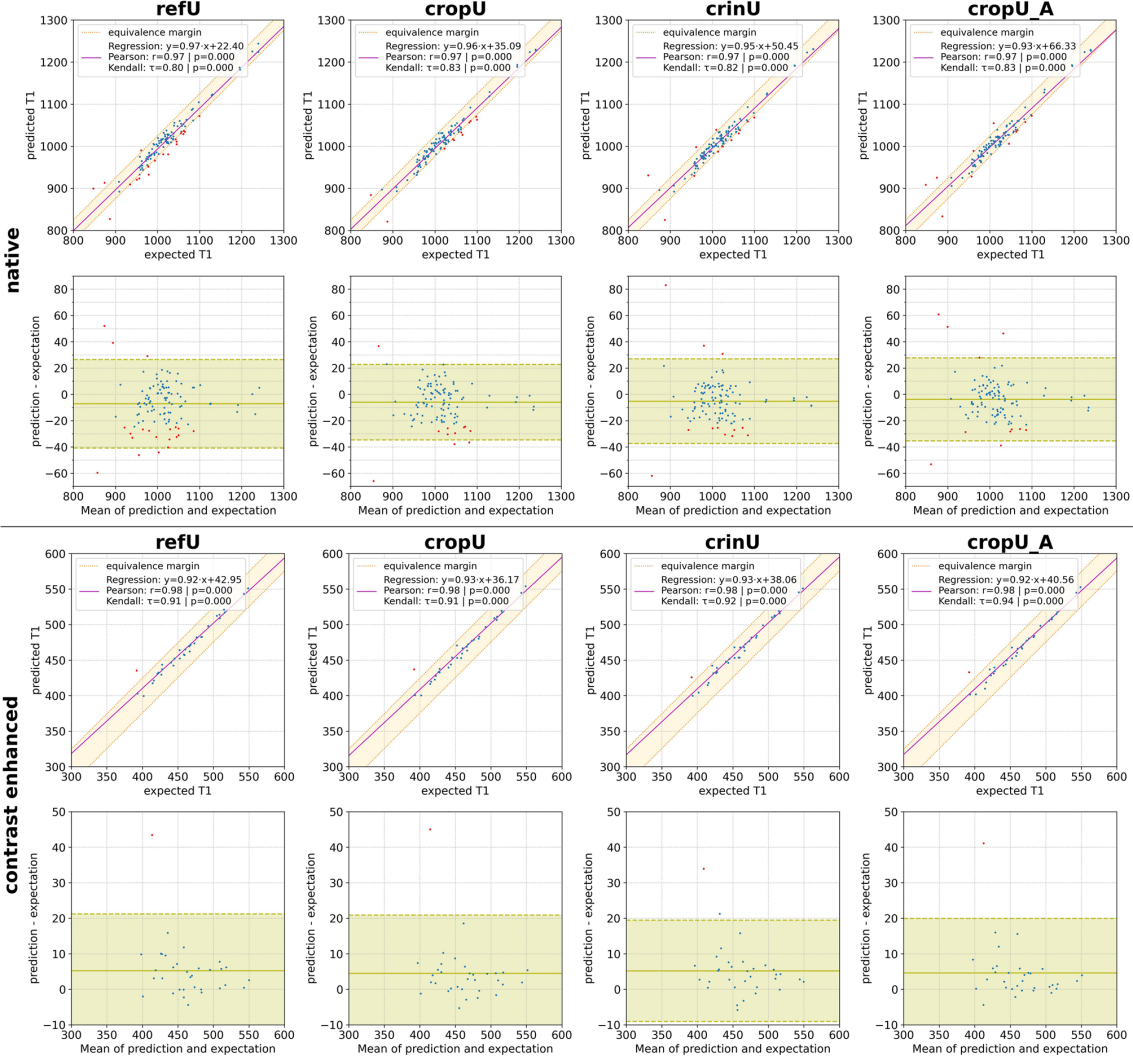
Quantitative results of the automated segmentation. The first column shows the quantitative results for refU, the second column for cropU, the third column for crinU and the fourth column for cropU_A. The upper block corresponds to native and the lower block to contrast enhanced data; respectively in each block the first row shows the correlation plot including the linear regression and the equivalence margin whereas the second row shows Bland–Altman-plots including the limits of agreement. Blue dots represent cases within the equivalence margin while red dots represent cases exceeding the equivalence margin.

Furthermore, all four pipelines showed a very strong linear correlation in native and CE test data, a strong monotonic correlation in the native test data and a very strong monotonic correlation in the CE data as shown in Table 2. The CoD was at least 94.09% such that the majority of the variation in the predicted average T1 time was explained by the variation of the targeted average left ventricular myocardial T1 time. Figure 5 shows the quantitative results as correlation- and Bland–Altman-plots in refU, cropU, crinU and cropU_A separately for native and CE test data. The plots indicate 20 native cases in refU, 11 native cases in cropU, 12 native cases in crinU, 11 native cases in cropU_A and 1 CE case in all pipelines that exceeded the limits of equivalence.

**Figure 6:**
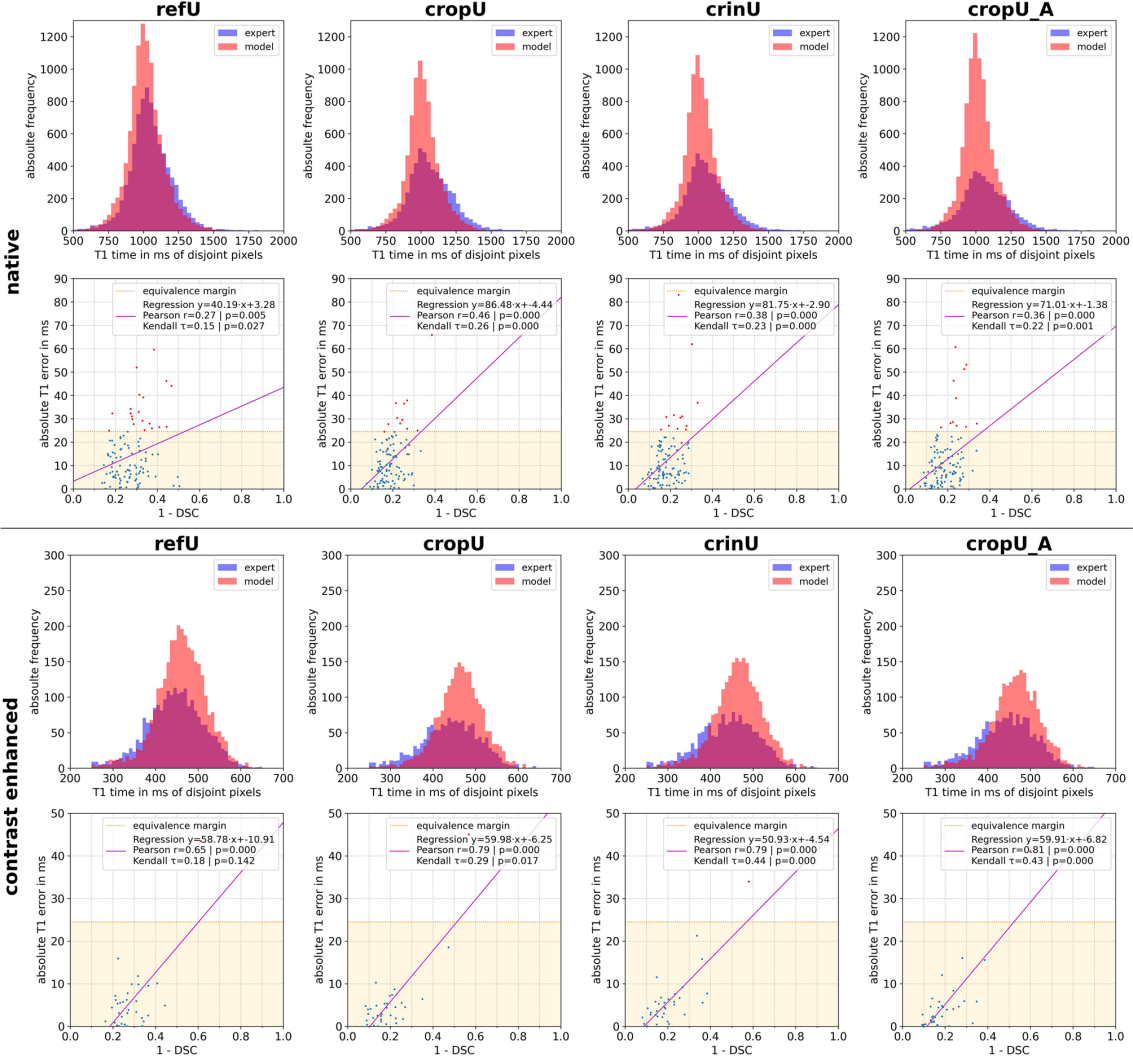
Coherence analysis of the automated segmentation. The first column shows the coherence analysis for refU, the second column for cropU, the third column for crinU and the fourth column for cropU_A. The upper block corresponds to native and the lower block to contrast enhanced data; respectively in each block the first row shows histograms of disjoint segmented pixel values of the expert ground truth and the pipeline model and the second row shows the correlation plot between Dice Similarity Coefficient (DSC) and the absolute T1 error including the linear regression. Blue dots represent cases within the equivalence margin while red dots represent cases exceeding the equivalence margin.

The Bland–Altman plots show that the limits of agreement differ only slightly between refU, cropU, crinU and cropU_A across the native as well as the CE test data. Further, the majority of those cases that exceeded the equivalence margin were underestimating the expected average T1 time. This was confirmed in the histograms of disjoint pixel values as shown in Fig. 6. The histograms show that false negative segmented pixels in the native test dataset tend towards higher T1 values whereas in the CE test dataset towards lower T1 values independent of any of the three pipelines. Individual outliers in the native histograms are outside of the plotted range, but occur rarely on values above 2000 ms.

Finally, the coherence analysis in Fig. 6 shows the relationship between the DSC and the absolute T1 error. While in the native test data refU showed a weak and all CASEG showed a moderate linear correlation, the linear correlation in the CE test data was moderate in refU and strong in all CASEG. In contrast to that, the rank order stability was only weak across all test data and pipelines except for a moderate stability in the CE test data for crinU and cropU_A. Facing the maximum Pearson correlation coefficient of 0.81, the maximum CoD only reached a value of 65.61% implicating that more than a third of the variation is not explained. Nonetheless, except for the rank order stability correlation in CE data for refU, both correlation indices are significant (p < 0.05) in any pipeline. Further, it shows that most cases in CASEG and almost half of the cases in refU that exceeded the 24.5 ms equivalence margin had a DSC above 70% which is assumed with a good geometric result^8^.

## Discussion

In CMR, the development of automated segmentation methods based on CNNs aims to substitute the necessity of an expert segmentation^30^. More complex network structures showed an improvement in segmentation quality^8,9,14^ while it is also known that the segmentation quality highly depends on the input data quality^31–33^. Hence, this study explored the impact of an upstream object detection as a quality enhancement of input data on the segmentation quality of parametric T1 maps. Our main findings show a significantly improved segmentation in the geometric domain when using an ODA as a pre-processing step in a CASEG pipeline with a U-Net based segmentation CNN while in the quantitative domain a consistent but statistically not significant improvement in the estimation of the average T1 times was observed.

### Dataset

The test dataset consisted of midventricular and basal slices only as those are recommended as stable slice location for a T1 map acquisition^4^. This differs from datasets described in the literature on automated segmentation methods for parametric T1 maps because either mid-ventricular slices only^9^ or the whole short axis stack were used^8,10^. Apart from this, a comparison of our models with those of the literature is restricted due to the lack of a common data basis in general. Furthermore, the ShMOLLI sequence as used in the literature gives different T1 mapping results than the used MOLLI sequence^34^. Therefore, a plausible comparison in the quantitative domain is limited.

### ODA

Object detection is used for the semantic understanding and localisation of objects in images^15^. While the classical use-cases of ODAs are the detection of multiple objects from numerous possible categories in a single image leading to highly complex network structures^15^, the ODA in the proposed CASEG pipelines had to find exactly one object from only one possible object class in an image. Hence, the use of a simple U-Net as ODA showed sufficiently good results for native and CE T1 maps in line with the results of Niu et al. who showed a DSC of 92.4 ± 3.6% in native CINE images for the left ventricular myocardial detection^16^. Our DSC results for the ODA were considerably above the 70% margin conventionally assumed as a good result^8^.

The ODA was used as the first step in a CASEG pipeline to increase the ratio of relevant pixels by cropping the image to a ROI representing an enlarged BB section. The applied magnification factor of 1.50 corresponds specifically to the used MOLLI T1 map short axis test dataset with our U-Net based ODA. As this factor is a freely adjustable hyperparameter in CASEG, an adaption is potentially necessary in other scenarios like different datasets or ODA networks. While the ODA network has a direct impact on the necessary magnification factor according to its prediction performance, two-dimensional quantitative data can be acquired in different orientations to meet the specific anatomy. Therefore, the optimal magnification factor is expected to be different in long axis views. Nonetheless, the stable DSC results across the magnification factor range of 1.30 to 2.50 for cropU and crinU showed similar performances even in out-of-optimum values for the magnification factor.

Although the ratio of relevant pixels was significantly (p < 0.05) increased in the ROI section compared to the original image, the result shows, that the majority of the pixels still belonged to background information. However, assuming a perfectly matched BB, this ratio could only be maximized to about twice the value gained, so that four out of five pixels would still belong to background information. The major reasons for this were the rather circular shape of the myocardium compared to the rectangular BB and the classification of the blood pool inside the myocardium as background information. The substantial variance in the ratio of relevant pixels within the 1.5 times enlarged BB as shown in the Supplemental Material S5 depended on the BB quality on the one hand and on the wall thickness of the myocardium on the other hand.

### CASEG

Although a complex network structure, analogous to the DoubleU-Net by Jha et al.^35^, could be used to integrate a whole CASEG pipeline into one network, the main idea of this work was to have a separated pre-processing step. Consequently, the ODA and the segmentation CNN in a CASEG pipeline are potentially interchangeable with other network structures. An alternative cropU structure with direct prediction of an enlarged BB omits the necessity of a magnification factor enlargement step at an equivalent outcome. Considering uncertainties during model training, cropU and cropU_A can be regarded as equivalent.

Our results emphasize that the upstream object detection improves the geometric segmentation quality in U-Net based automatic segmentation. Although the U-Net^12^ is a common CNN for medical image segmentation, one could expect that novel CNN architectures enable potential further performance gains^8,9,14^. The classical U-Net, as our refU pipeline, has been used in prior studies as a benchmark CNN as well. While the basic structure of those U-Nets is similar, implementation details such as hyperparameter settings potentially differs from the original and definitely from our refU network^8–10^ such that the performance comparability is limited. While Farrag et al. reached a DSC of 82.7% in native and 74.1% in CE T1 maps^10^, Puyol-Antón et al. showed a DSC of 78%^9^ and Hann et al. a DSC of 83.13%^8^ in U-Net based segmentation of native T1 maps. While the refU is inferior to the classical U-Nets in those studies for the native dataset, both CASEG pipelines, cropU and crinU, were able to align with those results. For the CE dataset, refU performance was inferior to the results of Farrag et al. while cropU and crinU were outperforming it. However, none of the pipelines could reach geometric results of 84% as in the probabilistic hierarchical segmentation network^9^ by Puyol-Antón et al. or 85% as in the quality control driven framework^8^ by Hann et al. potentially due to their more complex segmentation network structure. With respect to an intra-observer performance of 72% DSC and 15.61 mm HD in native data and 83% DSC and 9.03 mm HD in CE data the CASEG pipelines showed a robust geometric outcome compared to a human reader^13^. However, errors made by the automated segmentation are prone to be atypical as compared to a human reader^30^ such that the human segmentation is not necessarily substitutable by a completely unsupervised CASEG pipeline at the current stage.

Facing the quantitative domain of actual T1 values, no significant improvement in cropU and crinU compared to refU could be observed. This is at first glance counter-intuitive as a higher geometric accordance is assumed to coincide with a lower quantitative deviation. This was also shown in the coherence plot of Fig. 6 with a maximum CoD of 62.41% between DSC and the absolute T1 error underlining that an improved geometric result does not necessarily yield an improved quantitative result.

Taking into account, that the majority of the cases exceeding the equivalence margin in native T1 maps were underestimating the expert segmentation, the false negative segmented pixels belong to tissue that is assumed to contain blood. This agrees with the histograms in Fig. 6 and holds for the CE test data as well, as in CE blood has lower T1 values. Consequently, the border pixels are crucial as the impact of these disjoint pixels may be sufficient to impair improvements in the quantitative domain comparable to the significantly improved segmentation from a geometric point of view.

Comparing the quantitative results with literature values, the ME for the native dataset in refU, cropU and crinU lie in the published range of 4.6 ms^8^, 8 ms^10^ and 12.4 ms^9^ while the ME for the CE test data were worse than the 2 ms in the proposed segmentation method by Farrag et al. but much better than the ME in their comparative U-Net model with 37 ms^10^. However, the MAEs in the native dataset were slightly exceeding in all pipelines the result of 11.3 ms by Hann et al.^8^. Nonetheless, the CI of the quantitative results stayed in all cases within the intra-observer equivalence margin^11^.

As the segmentation quality depends on the input data quality^31–33^, we were able to show that the ODA in a CASEG enhancing the input data quality results in geometric improvements. However, partial volume effects along the endocardial contour may have an important negative impact on the quantitative outcome. In contrast to this study, the software cvi42 internally provides the possibility to use a kind of safety margin by moving the contours towards the middle of the myocardium by a predefined amount in order to compensate to a certain degree false positive segmented pixels at both borders. However, this procedure is not a standard option in all commercially available postprocessing solutions.

Finally, the CASEG as well as all other automated segmentation models found in the literature work with loss functions solely based on geometric agreement^8–10^ and neglect the quantitative domain. Therefore, the punishment for false positive segmented pixel during training of the models are equally independent of the actual T1 value. In conclusion it was shown in our study that an improved geometric congruence does not result in a significant minimization of T1 value deviation. Nonetheless, a significant (p < 0.05) but mainly only weak to moderate correlation between geometric congruence and quantitative deviation were shown. Additionally, the proposed network by Hann et al. shows the highest geometric congruence at lowest T1 deviation which suggests a DSC cutoff margin somewhere between 80 and 85% where the influence of the disjoint segmented pixels attenuate due to the high geometric overlap.

## Conclusion

The upstream object detection enables a significantly improved performance in the automated segmentation of parametric T1 maps from a geometric point of view compared to a standalone CNN. However, the quantitative measure could not be improved accordingly. Most likely the border pixels comprising partial volume effects between myocardium and blood play a key role in the discrepancy between geometric and quantitative results. As the quantitative domain is not represented in the training of the CNNs, segmentation of quantitative data like parametric T1 mapping may suffer from its absence. All in all, CASEG is well applicable for the improvement of segmentation tasks and this general approach may provide a viable extension to novel segmentation frameworks.

### Outlook

While this study showed the potential of an ODA in an automated segmentation pipeline, a future step could be the exchange of the U-Net based segmentation CNN in the CASEG with a higher performing architecture^8,9^ or more recent model adaptions^14^. This may provide an additional performance gain by the complex network structure in conjunction with the enhanced input data. Furthermore, it is worth investigating the combination of two CASEG pipelines for the segmentation of the myocardium by having one CASEG pipeline segmenting the blood pool area, which belongs to the endocardial contour, while the other CASEG pipeline segments the joined area of blood pool and myocardium, which represents the epicardial contour. The difference of both would return the myocardial segmentation and due to the hole-free segmentation masks, the ratio of relevant pixels may vastly increase. The latter enables the possibility of a better border definition.

Finally, one of the most crucial aspects in our study as well as in others is the definition of a loss function purely depending on the geometric concordance. A loss function taking both, the geometric as well as the quantitative deviation, into account would be preferable. As the domains are based on different physical units, the definition of such a loss function requires further investigation.

### Limitation

The used dataset is composed of available segmented parametric T1 maps coming from different studies and scanners. A prior selection in order to have equipartition in specific characteristics of the used dataset was omitted. Consequently, the dataset consisted of fewer CE images than native images. Furthermore, the results are limited to midventricular and basal slices only in the test dataset.

The hyperparameter setup of the U-Nets was chosen as the best performing one across multiple tested scenarios. However, this setup might be optimized for our specific dataset and not generalize well to others. Additionally, not all possible hyperparameter setups were tested due to its endless combination possibilities.

The magnification factor of the ODA predicted BB was based on and optimized for our test dataset. An adapted evaluation of the factor is potentially necessary when using different datasets or CNN structures. This work used U-Net based CNN models as case study of CASEG. However, reachable performance gains might be different in other network structures such as TransUNet^14^. The applied equivalence margin is the strictest one as it is based on an intra-observer variability. As the equivalence margin was defined on native T1 map data only, its application on CE T1 maps is questionable. Consequently, an adequate equivalence margin for CE T1 maps or in its usage in a ECV map is currently missing and were substituted in this study by the analysis of the CV.

## Supplementary Information

Supplementary Information 1.

Supplementary Information 2.

## Data Availability

The trained models and used dataset are available on request in an anonymized manner by contacting the first (DV: darian-steven.viezzer@charite.de) or last (JSM: jeanette.schulz-menger@charite.de) author. The source code can be accessed via GitHub under the URL: https://github.com/DSV-CUB/CASEG or in the Supplemental Material S7.
