# Supplementary material for: Introduction of a cascaded segmentation pipeline for parametric T1 mapping in cardiovascular magnetic resonance to improve segmentation performance
Source: Sci Rep. 2023 Feb 6;13:2103. doi: 10.1038/s41598-023-28975-5 (PMC9902617; doi:10.1038/s41598-023-28975-5)

**Introduction of a cascaded segmentation pipeline for parametric T1 mapping in cardiovascular magnetic resonance to improve segmentation performance**

Darian Viezzer^1,2^, Thomas Hadler^1,2^, Clemens Ammann^1,2^, Edyta Blaszczyk^1,2^, Maximilian Fenski^1,3^, Thomas Hiroshi Grandy^1,3^, Jens Wetzl^4^, Steffen Lange^5^, Jeanette Schulz-Menger^1,2,3,*^

^1^ Charité Universitätsmedizin Berlin, Working Group on Cardiovascular Magnetic Resonance, Experimental and Clinical Research Center, a joint cooperation between the Charité Universitätsmedizin Berlin and the Max-Delbrück-Center for Molecular Medicine, Berlin, Germany

^2^ DZHK (German Centre for Cardiovascular Research), partner site Berlin, Berlin, Germany

^3^ Helios Hospital Berlin-Buch, Department of Cardiology and Nephrology, Berlin, Germany

^4^ Siemens Healthcare GmbH, Erlangen, Germany

^5^ Hochschule Darmstadt (University of Applied Sciences), Faculty for Computer Sciences, Darmstadt, Germany

* Corresponding author, E-Mail: jeanette.schulz-menger@charite.de

S1: Processing pipeline for refU and the alternative cropU (cropU_A). Convolutional neural networks (CNNs) are used for the segmentation of the myocardium as tissue of interest. While refU directly uses the input image, cropU_A uses the region of interest image section that belongs to the predicted enlarged bounding box from an object detection algorithm (ODA).


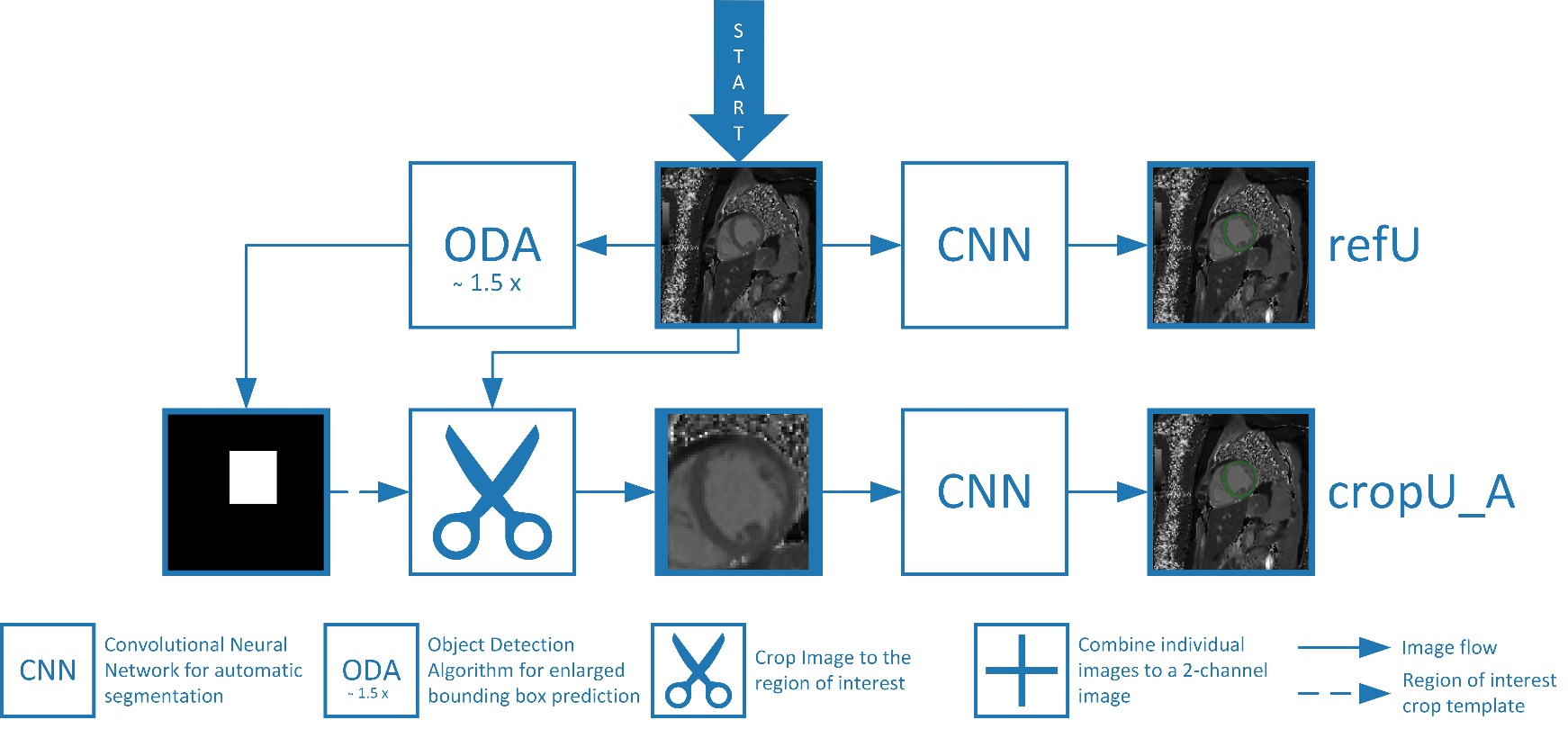


S2: U-Net Model structure. The original input image, denoted as orange, is resized towards the U-net specific input size. The blue boxes represent the feature maps of the model with their specific size. The output is back transformed to the size of the original input image, denoted in green. All arrows represent an action task on the image or feature map.


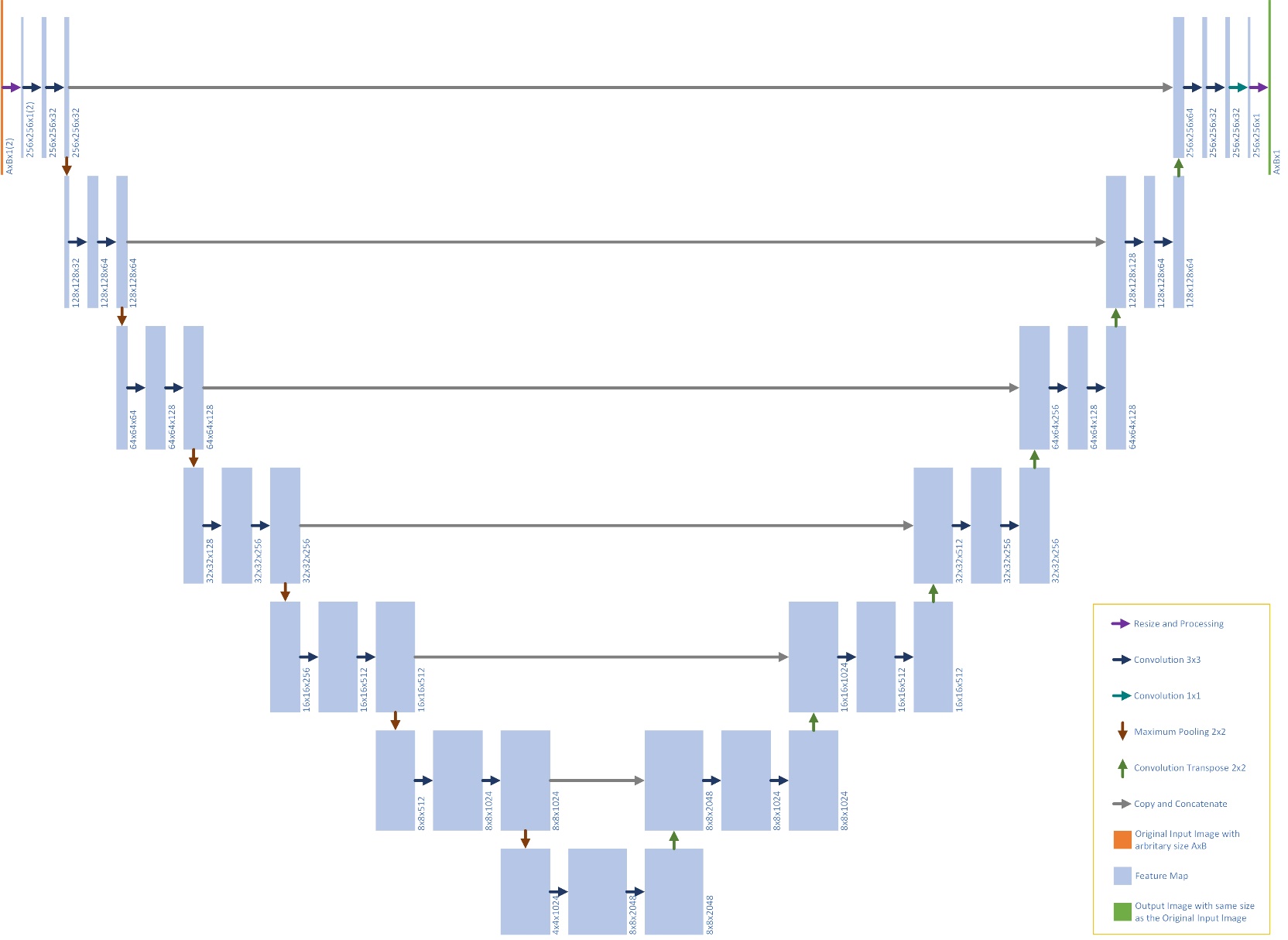


S3: Overview of Python Packages that are used in this study.

| **Package** | **Version** |
| --- | --- |
| GDAL | 3.4.1 |
| geopy | 2.2.0 |
| h5py | 2.10.0 |
| keras | 2.7.0 |
| matplotlib | 3.5.2 |
| mock | 4.0.2 |
| numpy | 1.21.5 |
| opencv-python | 4.4.0.42 |
| openpyxl | 3.0.9 |
| pandas | 1.3.4 |
| Pillow | 7.2.0 |
| pip | 20.1.1 |
| polyline | 1.4.0 |
| pydicom | 2.2.2 |
| rasterio | 1.2.10 |
| scikit-fuzzy | 0.4.2 |
| scikit-image | 0.18.1 |
| scikit-learn | 1.0.2 |
| scipy | 1.4.1 |
| Shapely | 1.8.0 |
| statsmodels | 0.13.0 |
| tensorflow | 2.7.0 |
| tensorflow-gpu | 2.7.0 |
| XlsxWriter | 3.0.1 |
| xlwings | 0.25.0 |

S4: Average Dice Similarity Coefficient (DSC) results for the test dataset segmentation in cropU and crinU with respect to the magnification factor for the bounding box enlargement. The red dotted line at 1.44 refers to the optimum magnification factor value by calculation in order to guarantee all BBs include the complete left ventricle as tissue of interest.


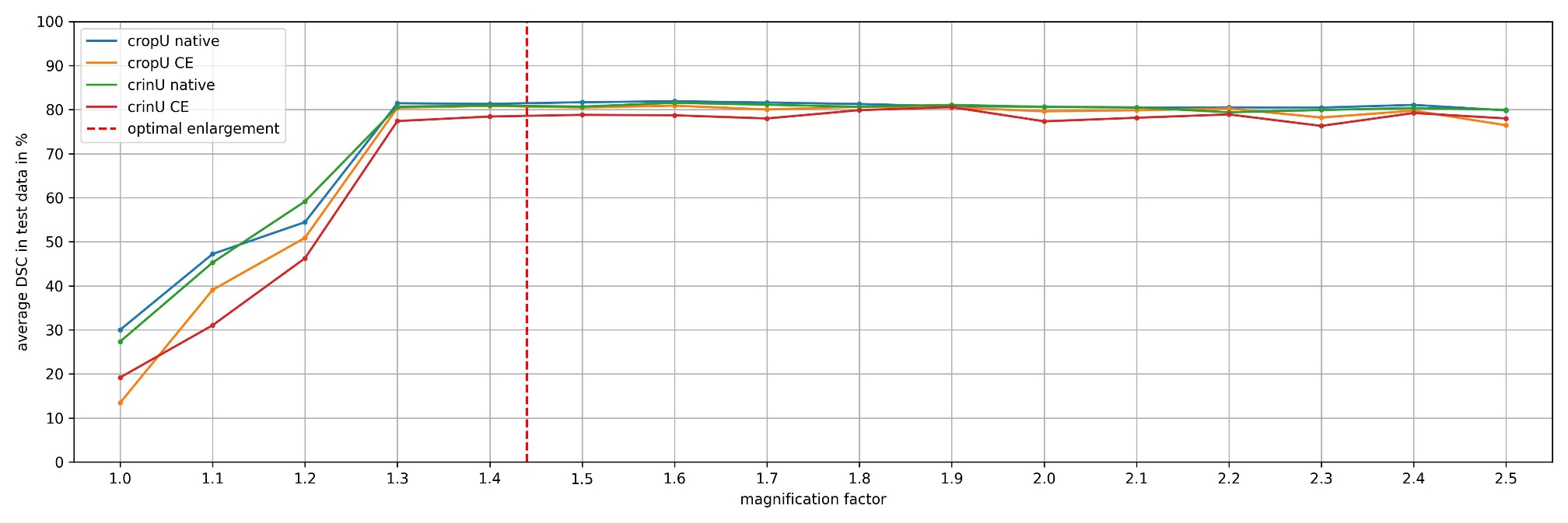


S5: Ratio of relevant pixels. The first column shows the ratio for the training, the second column for the validation and the third column for the test dataset ratio. The upper block corresponds to native and the lower block to post contrast agent data; respectively in each block the first row shows the ratio in the original image and the second row shows the ratio in the cropped image section that corresponds to the 1.5 times enlarged bounding box.


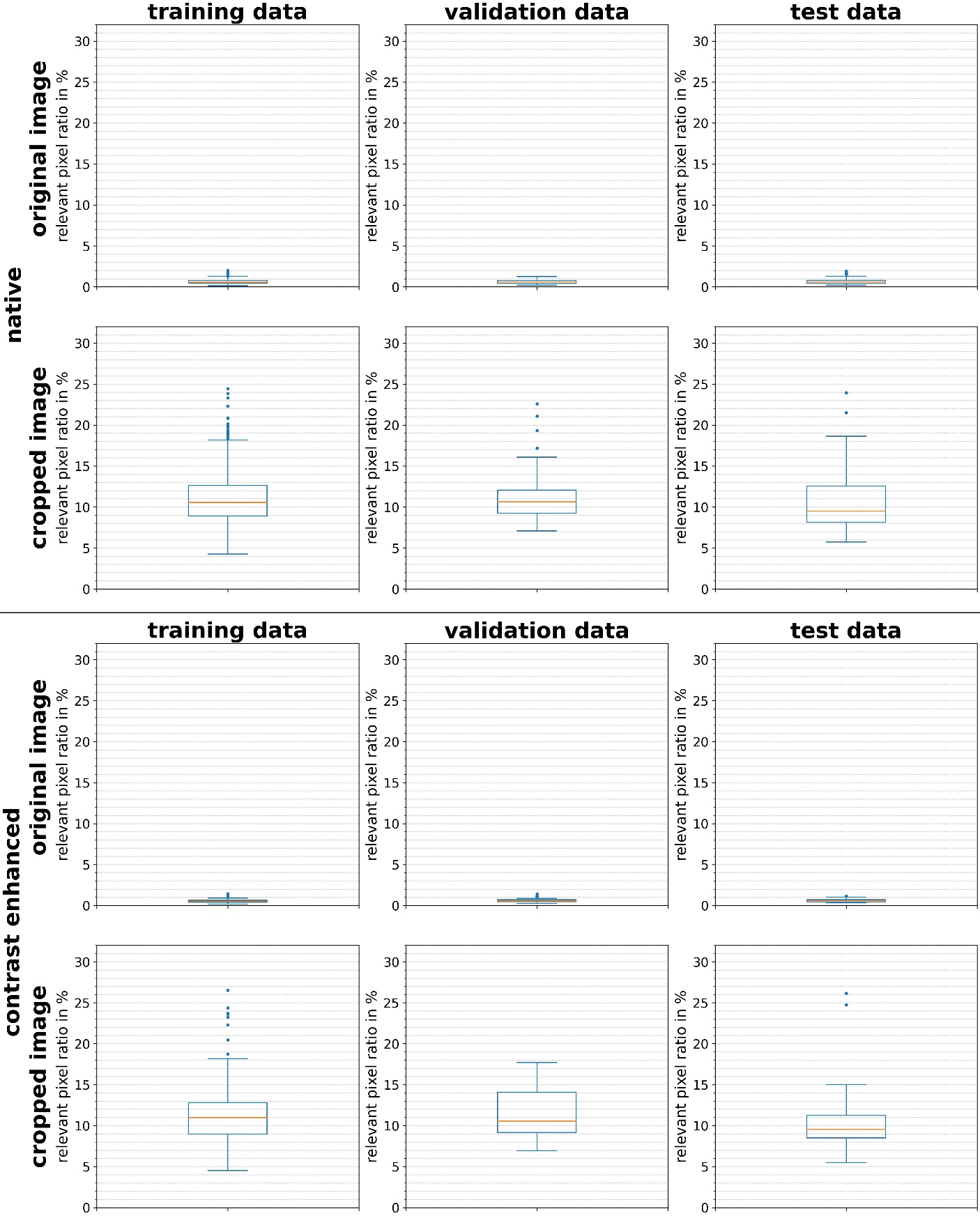


S6: Equivalence margin plot. The first column shows the equivalence margin for refU, the second column for cropU,the third column for crinU and the fourth column for cropU_A. The upper block corresponds to native and the lower block to contrast enhanced data.


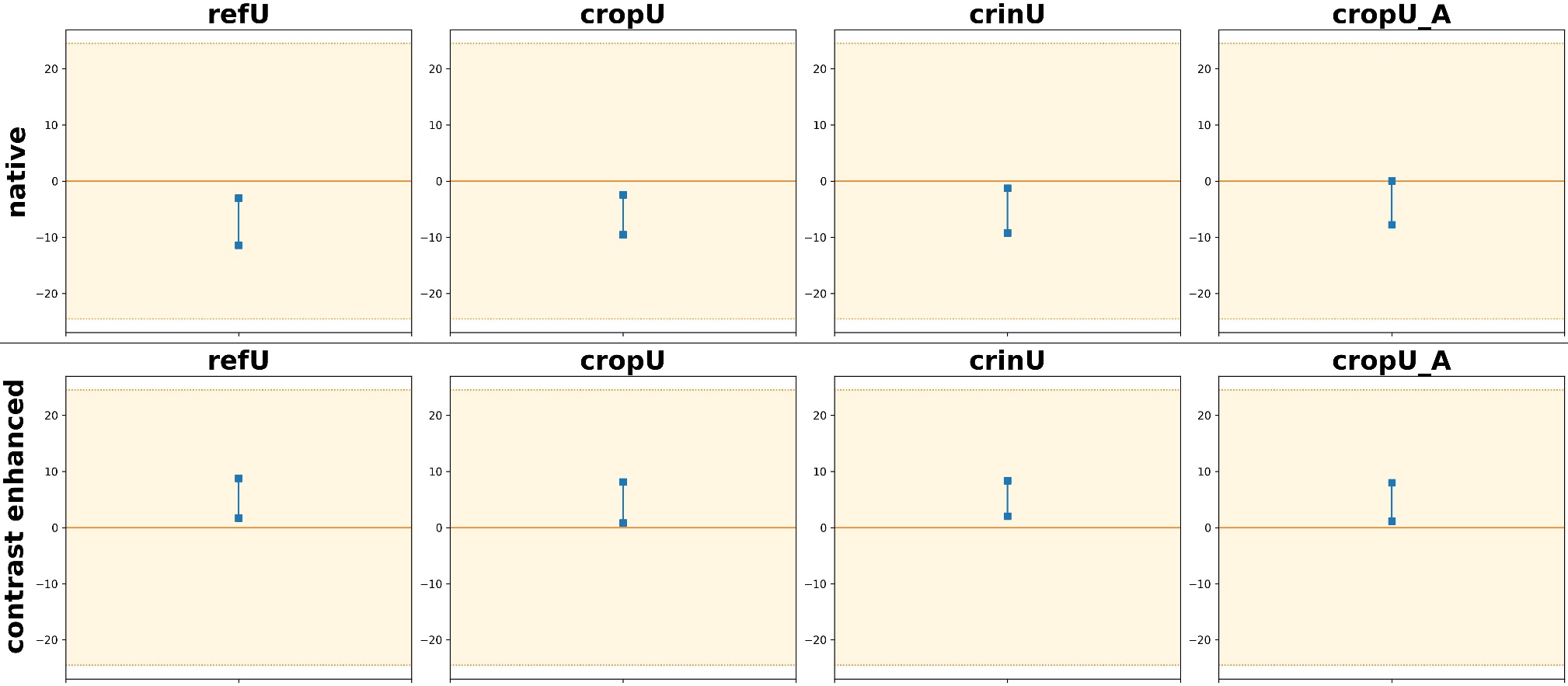

Supplement: Supplementary file 1 — Supplementary Information 1. [file 41598_2023_28975_MOESM1_ESM.docx]
